# Development of a 3D Underground Cadastral System with Indoor Mapping for As-Built BIM: The Case Study of Gangnam Subway Station in Korea

**DOI:** 10.3390/s151229833

**Published:** 2015-12-09

**Authors:** Sangmin Kim, Jeonghyun Kim, Jaehoon Jung, Joon Heo

**Affiliations:** 1 School of Civil and Environmental Engineering, Yonsei University, 50 Yonsei-ro, Seodaemun-gu, Seoul 120-749, Korea; netgo82@yonsei.ac.kr (S.K.); jhkim2014@yonsei.ac.kr (J.K.); lionheart_kr@yonsei.ac.kr (J.J.); 2 Department of Photogrammetry, University of Bonn, Nussallee 15, Bonn 53115, Germany; j.jung@igg.uni-bonn.de

**Keywords:** 3D underground cadastral system, indoor mapping, as-built BIM, terrestrial laser scanning

## Abstract

The cadastral system provides land ownership information by registering and representing land boundaries on a map. The current cadastral system in Korea, however, focuses mainly on the management of 2D land-surface boundaries. It is not yet possible to provide efficient or reliable land administration, as this 2D system cannot support or manage land information on 3D properties (including architectures and civil infrastructures) for both above-ground and underground facilities. A geometrical model of the 3D parcel, therefore, is required for registration of 3D properties. This paper, considering the role of the cadastral system, proposes a framework for a 3D underground cadastral system that can register various types of 3D underground properties using indoor mapping for as-built Building Information Modeling (BIM). The implementation consists of four phases: (1) geometric modeling of a real underground infrastructure using terrestrial laser scanning data; (2) implementation of as-built BIM based on geometric modeling results; (3) accuracy assessment for created as-built BIM using reference points acquired by total station; and (4) creation of three types of 3D underground cadastral map to represent underground properties. The experimental results, based on indoor mapping for as-built BIM, show that the proposed framework for a 3D underground cadastral system is able to register the rights, responsibilities, and restrictions corresponding to the 3D underground properties. In this way, clearly identifying the underground physical situation enables more reliable and effective decision-making in all aspects of the national land administration system.

## 1. Introduction

The International Federation of Surveyors (FIG) defines a cadaster as follows: it “is normally a parcel based and up-to-date land information system containing a record of interests in land. It usually includes a geometric description of land parcels linked to other records describing the nature of the interests, and ownership or control of those interests, and often the value of the parcel and its improvements” [1]. In short, an important function of the cadastral system is to support the protection of ownership and provide spatial information by registering physical objects. Conventionally, the system is not linked to 3D properties but is mainly related to only 2D surface parcels themselves.

However, due to population growth and shortage of available land, especially in urban areas, property development has been conducted extensively through both above-ground and underground spaces. This has incurred a variety of problems related to the registration and management of 3D properties based on a 2D cadastral system [2]. Thus, registration of 3D property rights has become an important issue in the cadastral domain, because it is a fundamental function for protection of ownership, property tax assessment, land use, and land management systems [3,4,5]. As such, it is used directly by land registries, certified public appraisers, real estate agents, city planners, and landowners. Thus too, validated 3D cadastral data has to be established and provided. Consequently, 3D cadastral system research and development continue to be critical land management issues, and Korea is no exception in this regard.

The issues impacting 3D underground property in Korea can be summarized as follows: boundary disputes from the ambiguity of the definition of scope of justifiable profit, administrative confusion in implementing the registration, management and utilization of underground space, double compensation problem, and the lack of adequate plans and systematic registration for underground architectures and civil infrastructures. These problems are occurring under the lack of specific laws and regulations for registering and managing underground properties [6,7,8,9,10]. In this light, the main purpose of the present study was to devise new concepts for the 3D underground cadastral system by adopting the indoor mapping method used for as-built Building Information Modeling (BIM) [11,12,13]. This investigation originated from our preceding studies on productive high-complexity 3D city modeling of building exteriors and productive modeling for development of as-built BIM of indoor structures [14,15]. These were mainly focused on the modeling of building components in the construction management domain. However, in the present paper, a method of 3D underground cadastral mapping based on indoor mapping for as-built BIM is proposed. This method represents a new-concept mapping framework for 3D underground properties in the cadastral domain. The remainder of this paper is structured as follows. In Section 2, a review of the literature on the 3D cadastral system and indoor mapping for as-built BIM is conducted. In Section 3, an overall 3D underground cadastral system development procedure consisting of the following four steps is proposed: (1) geometric modeling of underground construction components; (2) as-built BIM in Revit software; (3) accuracy assessment of as-built BIM; and (4) creation of 3D underground cadastral maps for the 3D underground property. In Section 4, the implementation of the proposed method in a real underground infrastructure for creation of 3D underground cadastral maps using as-built BIM data is presented. In Section 5, conclusions are drawn and future work is anticipated.

## 2. Related Work

The 3D cadastre “registers and gives insight into right and restrictions not only on 2D parcels, but also on 3D property units” [16]. Required for the establishment and management of a 3D cadastre are 3D property registration laws, 3D data acquisition methods, 3D spatial database management systems, and a functional 3D visualization platform [17]. 3D data acquisition and efficient data processing related to registration of 3D properties, not only for above-ground but also for underground spaces, are crucial land administration steps in the determination and management of legal information with respect to rights, responsibilities, and restrictions.

In numerous cadastral-domain studies, a variety of methodological approaches, namely range-based modeling, image-based modeling, and some integrated approaches, have been employed for mapping and registration of 3D properties [18]. The most widely utilized method for acquiring 3D properties is terrestrial laser scanning, owing to its relative ease of application to 3D feature extraction [14,15,19,20,21,22,23,24,25,26,27]. Image-based modeling offers advantages including lower cost, faster data acquisition, and less manpower required for extraction of land-parcel boundaries and external building components [28,29,30,31,32,33]. Its disadvantages, however, are its unsuitability for irregularly shaped or complex construction, low-light conditions, and oblique-viewing-angle sites [34]. Over the past decade, integrated range-based and image-based modeling have been developed by means of aerial laser scanning and aerial photogrammetry [35,36,37,38] as well as the integration of terrestrial laser scanning and terrestrial photogrammetry [19,39,40,41].

Most of these methods, unfortunately, are designed for extraction and reconstruction only of external building information; that is, they are effective only for 3D above-ground cadastral systems, and are not well customized to 3D underground cadastral systems. Interior mapping for 3D underground cadastre is opposite to exterior mapping for 3D above-ground cadastre. In that context, interior mapping, which is officially termed “indoor mapping”, is an indispensable step for the representation of various types of 3D underground properties.

Indoor mapping, depicting fundamental building components such as floors, walls, and ceilings, is becoming more commonly used for as-built BIM. It has emerged as a powerful tool for compiling information on actual building conditions and modeling accordingly [11,12,13,27]. With regard to the operation and maintenance (O&M) of architectures and civil infrastructures, it has been proven that as-built BIM can improve the efficiency of building and facility management by providing for building safety, building lifespan, sustainable management, space management, maintainability, and control of energy consumption [42]. However, the procedure of 3D as-built BIM for buildings and facilities remains heavily reliant on manual processing, not only of geometric but also semantic information [22,43,44]. In order to overcome the inefficiencies of manual geometric processing, in 3D as-built BIM, photogrammetry and terrestrial laser scanning have become more commonly utilized for geometric data acquisition of 3D features than conventional surveying equipment such as total station and measurement tapes [45]. In the as-built BIM field, photogrammetry can be used to generate 3D building reconstructions based on image processing and computer-vision methodologies. Nonetheless, it has limitations, especially for building interiors, due to problems including the lack of feature extraction from non-textured surfaces, complex geometric conditions, and lighting conditions [45,46].

Terrestrial laser scanning can generate point clouds with a large number of points containing abundant 3D location information. It has some disadvantages, however, such as cost, maintenance, and, not least, the size of the acquired point cloud data. On the other hand, it is very accurate, offers high resolution, and is not sensitive to light conditions [47]. Most notably, it can handle sharp corners and edges, which is critical for as-built BIM [48]. For that reason, terrestrial laser scanning is considered the most suitable method—and indeed is the prevailing choice—for modeling of complex geometric buildings [13,18,45,49,50,51].

In this research, a framework for 3D underground cadastral system based on data acquisition with terrestrial laser scanning and 3D underground property mapping with an indoor mapping method designed for as-built BIM is proposed.

## 3. Proposed Method

### 3.1. Overviews

**Figure 1:**
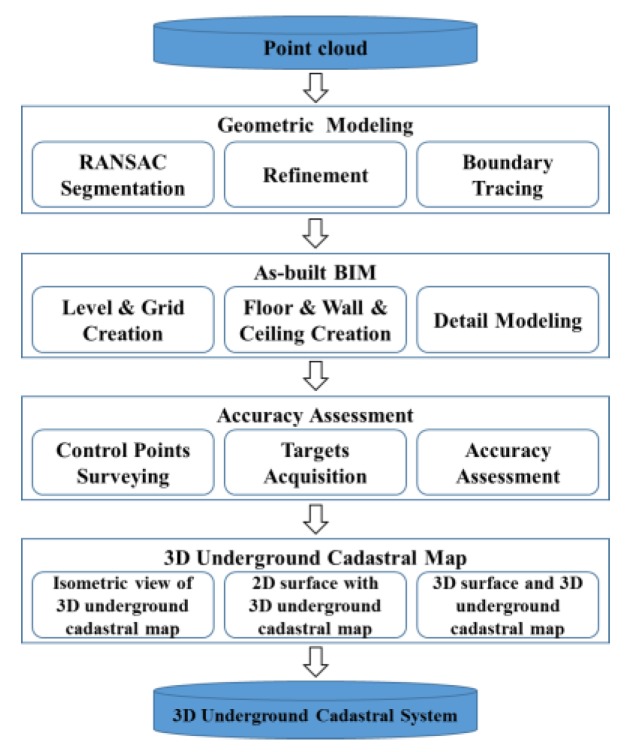
Overall procedure of proposed method.

A 3D underground cadastral system based on indoor mapping methodology developed for as-built BIM is proposed. The four data processing steps are as follows: (1) geometric modeling of underground properties; (2) as-built BIM in Revit software; (3) accuracy assessment; and (4) creation of 3D underground cadastral map based on as-built BIM. In the geometric modeling step, the plane components are extracted from point clouds by RANdom SAmple Consensus (RANSAC) segmentation [52,53,54]. Then, the refinement process is applied for removal of noise and boundary tracing. In the as-built BIM step, the geometric modeling is imported into BIM software to create as-built BIM, after which an accuracy assessment is conducted. In this accuracy assessment step, control-points surveying and targets acquisition are conducted to evaluate the quality of as-built BIM. In the 3D underground cadastral map creation step, an isometric view of the 3D underground cadastral map, a 2D surface parcel with footprints of the 3D underground cadastral map, and a 3D surface and 3D underground cadastral map are produced in order to register 3D underground properties. Figure 1 illustrates the method’s overall procedure.

### 3.2. Geometric Modeling

The methodology of geometric modeling, derived in our previous research, consists of three phases: (1) segmentation; (2) refinement; and (3) boundary tracing [14,15]. In the segmentation phase, plane components are identified and directly extracted from point clouds in the polygon type. This is the initial and most important step in 3D as-built BIM. In the present study, the RANSAC algorithm [54], among a variety of segmentation algorithms including Hough transform [55], Gaussian sphere [56], Expectation Maximization (EM) [57], tensor voting [58], region growing [59], and others, was selected for identification and extraction of plane components due to its ability to extract a variety of different shapes of planes, not to mention the fact that it is the most widely used and robust segmentation method with respect to noisy data [53,60,61,62]. RANSAC was originally proposed for robust fitting of a model from a dataset containing significant gross errors. It consists of inliers that can be represented by a set of model parameters and outliers that do not fit the model. RANSAC includes a hypothesis step and a test step. To achieve the best results, the two steps are iteratively processed until the number of iterations (*k*) is reached. Iteration (*k*) can be described by Equation (1):

(1) $$k=\frac{\log(1-p)}{\log(1-w^{n})}$$

where p is the probability that all randomly selected points are inliers, n is the number of samples selected in the hypothesize step, and w is the likelihood ratio that a point belongs to the best plane. The Root Mean Squared Error (RMSE) can be used to determine the best planes. The RMSE can be described by Equation (2):

(2) $$RMSE=\sqrt{\sum_{i=1}^{N}{\left(\frac{\left|ax_{i}+by_{i}+cz_{i}+d\right|}{\sqrt{a^{2}+b^{2}+c^{2}}}\right)}^{2}\cdot \;\frac{1}{N}}$$

where (*x*, *y*, *z*) are Cartesian coordinates of a point, (*a*, *b*, *c*, *d*) is a parameter vector on the 3D plane, and *N* is the number of points in set *X*.

Based on the RANSAC segmentation method, a variety of planes are extracted. However, noisy points, which are mis-classification results, can also be extracted through RANSAC processing. In order to solve this problem, a labeling method is used to filter erroneously segmented planes. The model plane with inlier points, extracted by RANSAC, is rotated and projected onto a binary image on the x-y plane.

**Figure 2:**
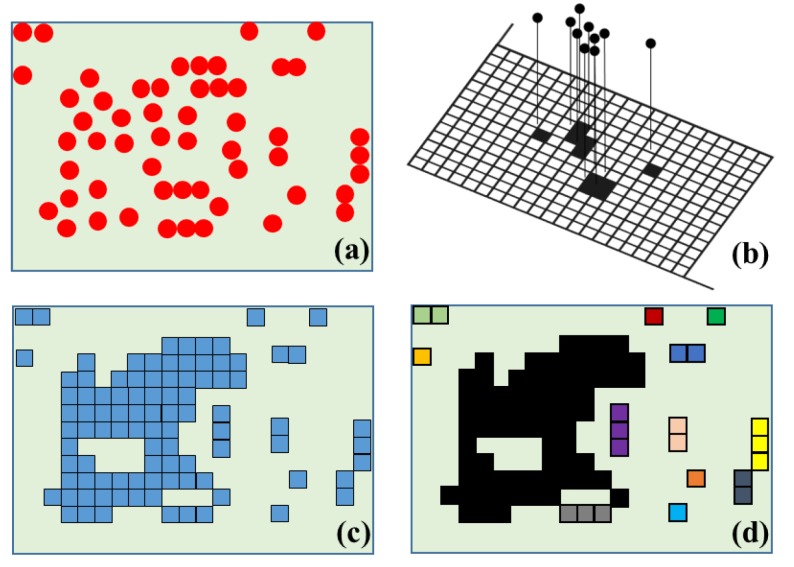
Conceptual illustration of the refinement process: (**a**) Inlier points of model plane; (**b**) Projection of points on plane; (**c**) Rasterized image; (**d**) Result of image labeling (gray-colored area is integrated into the largest black-colored area; the other colored areas, such as yellow, green, blue, *etc.*, are considered to be noise). (Figure is adopted from [15]).

This binary image can be divided into two categories, occupied pixels (as “1”) and non-occupied pixels (as “0”). In this way, the labeling method determines the pixels’ interconnectivity. Then, the connectivity of each plane component is labeled and its pixels are counted [63]. Finally, the largest area of the binary image remains, and the others, considered as noise, are removed. Figure 2 provides a conceptual illustration of the refinement process.

**Figure 3:**
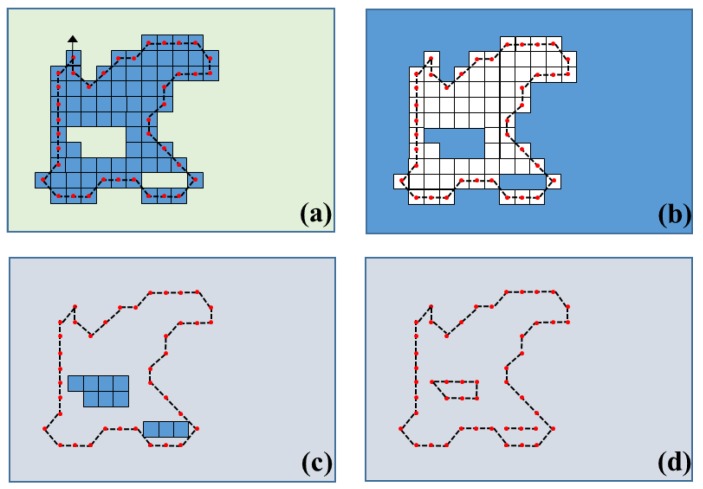
Boundary tracing and hollow objects detected by scheme: (**a**) Boundary tracing; (**b**) Inversion of binary raster image; (**c**) Hollow area detection by labeling; (**d**) Boundary tracing of hollow area (Figure is adopted from [15]).

In the boundary tracing step, the boundary pixels are traced out only from the occupied pixels. In order to do that, it begins with from the top left occupied pixel in the binary image. Then, the next adjacent boundary pixels are checked to link between each pixel through consistent processing in the clockwise direction. In order to create a boundary of hole components including gates and open doors, the original binary image has to be inversed to represent these kinds of spaces as 1 and the others, which are the original binary image spaces, as 0. Then, the same processes of labeling and boundary tracing are conducted for each of the empty and opened spaces. Finally, traced-out boundary features including the extracted plane are produced and returned to the original coordinates system inversely. Figure 3 illustrates the concept of boundary tracing.

For the refinement and boundary tracing steps, the grid cell size of the binary image has to be carefully defined for a series of raster processing. It directly affects the result of geometric modeling from point clouds. According to the recommendations of the previous study and several experiments for the project site, 0.05 m is chosen as the optimal grid cell size [14,15].

### 3.3. As-Built BIM

Based on the processing outlined above, 3D boundary lines and the remaining points, produced from the 3D point cloud, provide sufficient support to the modeler for efficient identification and modeling of building components. Compared with conventional direct 3D modeling, the proposed method increases productivity and enhances quality. Because the data size is reduced to 5% of the point cloud size or less, 3D boundary lines with topological relationships of plane features can be used mainly as reference data for the 3D as-built modeling. Moreover, without preprocessing, 3D modeling system failure can occur due to the gigantic size of point cloud data [14]. In this study, Autodesk Revit 2014 [64] was selected for the 3D as-built modeling. Finally, by using the geometric modeling method, the productivity of 3D as-built BIM is improved, especially by reducing the necessary human effort [14,15].

### 3.4. Accuracy Assessment

To evaluate the quality of the as-built BIM, accuracy assessment is conducted by comparison with designated target points. In this study, accuracy assessment consisted of three phases: (1) control-point surveying; (2) target-coordinate acquisition; and (3) accuracy assessment.

Control-point surveying establishes a consistent and accurate network of physical features on the ground, and defines the geographic positions of project sites. This network consists of a vertical reference network and a horizontal reference network. The vertical reference network is established using leveling, and the horizontal reference network by using total station to tie all of the control points by angle and distance. Then, target-coordinate acquisition is conducted by total station, which is radial surveying based on established control points. In order to accomplish that, target points have to be well-defined points such as crossing edges of building components, and they have to be evenly-distributed for overall geometric accuracy. Finally, the acquired target points are assessed according to the Euclidean average distance error, Root Mean Square Error (RMSE), and Spherical Accuracy Standards (SAS).

### 3.5. Concepts of 3D Underground Cadastral Map

The fundamental purpose of the cadastral system is to support the registration of a title with a legally binding digital cadastral map [65]. Currently, most of cadastral systems are still maintained in the 2D cadastral map format [5,66]. For that reason, this study suggests three types of 3D underground cadastral mapping to identify and prove the physical status of 3D underground parcels with (1) an isometric view of the 3D underground cadastral map [17,67,68]; (2) a 2D surface parcel with footprints of the 3D underground cadastral map [2,69,70]; and (3) a 3D surface and 3D underground cadastral map [71,72,73,74,75].

The isometric view of the 3D underground cadastral map is used to describe the geometric information of a 3D underground parcel separately from the surface parcel. This map describes the volume of 3D underground property in the cadastral system. It is able to register spatial information details such as the coordinates of boundary corner points, boundary distances, planimetric areas, and volumes of given underground property. Thus, it can deal with a variety of information, including rights, restrictions, and responsibilities, related to 3D underground property.

A 2D surface parcel with footprints of the 3D underground cadastral map is used to represent the 3D underground property with corresponding surface parcels. It is able to verify the ownership between underground parcels and surface parcels in order to prevent vertical land disputes and to provide better public land administration. Additionally, it can incorporate a 3D underground cadastral system with an existing 2D cadastral map, because most of the current land management systems, such as the Korea Land Information System (KLIS), the Urban Planning Information System (UPIS) [76], and the Land Use Regulations Information system (LURIS), are based on 2D cadastral maps [77].

A 3D surface and 3D underground cadastral map are used to represent the location of 3D underground properties with regard to real-world situations. They are able to register and manage depth information, which represents the difference between 3D surface and 3D underground property. Through accurate representation of 3D underground property, mapping can deal with land administration issues including underground ownership, underground compensation, and underground taxation. Thereby, it can support the various stakeholders in establishing, supporting, and maintaining their legal rights over 3D underground property.

## 4. Application

The proposed methodology described in the previous section was implemented in a real underground infrastructure in order to create a 3D underground cadastral map.

### 4.1. Project Site and Data Acquisition

The project site is the Gangnam subway station on Seoul Metro subway line 2 in Seoul, Korea. Gangnam subway station is the busiest subway station among the 119 stations in Seoul. Its average daily traffic is 135,642 people/day [78]. This station consists of two floors: the first-floor underground shopping mall includes circulation space, a number of stores, public spaces such as lounges and restrooms, and facility management spaces such as mechanical facility rooms, storage rooms, management offices, and disaster prevention facilities; the second underground floor is the subway platform. In this study, due to the limitations of as-built surveying (such as restriction of access to facility management space and commercial space under the leasehold), the data acquisition focused mainly on the circulation space. For the rest of Gangnam subway station, floor plans were principally utilized.

**Table 1:** Point cloud acquisition and test environment specifications.

Categories	Specifications
Target Study Area	Gangnam subway station: underground shopping center and subway station platform (Gangnam-gu, Seoul, Korea)
Extent of Subway Station (Along Centerline)	Length = 254.116 m
	Width = 177.5 m
	Height = 7.05 m
Type of Terrestrial Laser Scanner	Scanner model: Leica Scan Station P20
	3D position accuracy: 3 mm at 50 m, 6 mm at 100 m
	Linearity error: ≤1 mm
	Angular accuracy: 8″ (horizontal/vertical)
Laser Scanning Data	Number of stations: 171 stations (1st floor: 126, 2nd floor: 45)
	Data size: 4.85 GB
	Number of points: 106.7 million
Coordinate System	Project coordinate system: Korea 2000 central belt 2010
	Datum: Korea 2000 (KGD2002)
	Ellipsoid: GRS1980
	Projection: Transverse Mercator
Processing Environment	CPU: Intel^®^ Core™ i7-4790 CPU@3.60 GHz
	RAM: 32.0 GB
	OS: Windows 7 64-bit
Software	Point cloud processing: Matlab 8.1.0
	As-built Modeling: Autodesk Revit 2014

**Figure 4:**
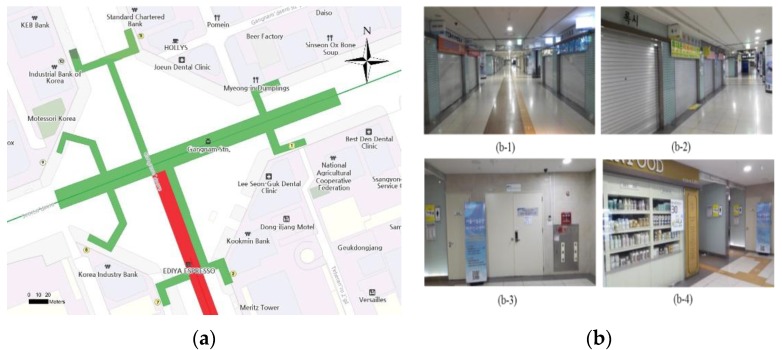
Project site: (**a**) study area; and (**b-1**, **2**, **3**, **4**) Gangnam subway station underground shopping center; (**b-1**) circulation space; (**b-2**) store space; (**b-3**) facility management space; and (**b-4**) public restroom.

Terrestrial laser scanning was conducted in the study area using Leica P20. In order to register point clouds of the Gangnam subway station into a Korean coordinate system, Global Position System (GPS) surveying was conducted for six control points that were set up at entrances of the subway station. The high-quality control points were also scanned and used to register into terrestrial laser scanning data of Gangnam subway station. The specifications of the subway station, coordinate system, surveying equipment, and methods are summarized in Table 1. Figure 4 illustrates the location information on the project site with a map and photos.

### 4.2. Segmentation and Geometric Modeling

**Figure 5:**
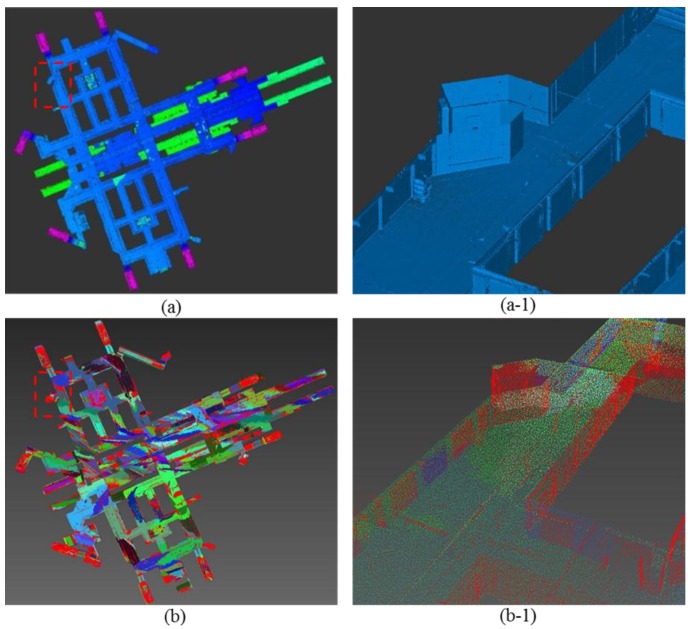

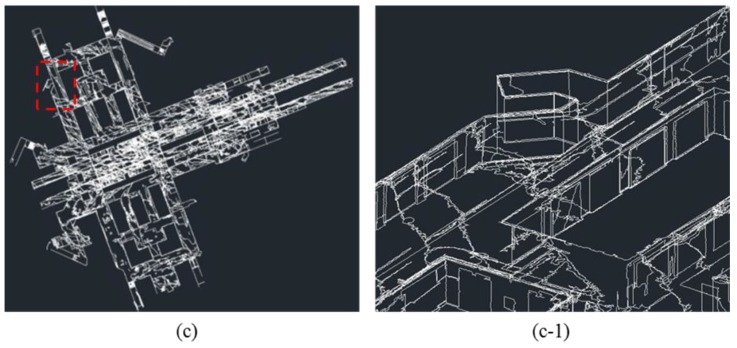
The result of each step in the processing: (**a**) Scanned point cloud; (**a-1**) Detailed description of (**a**); (**b**) Segmented planes; (**b-1**) Detailed description of (**b**); and (**c**) Geometric modeling; (**c-1**) Detailed description of (**c**).

The results for each step in the processing are illustrated in Figure 5. Figure 5a shows the registered point clouds, and Figure 5a-1 shows the scanned point cloud of the selected area indicated by the dotted lines in Figure 5a. Figure 5b shows the results for each of the segmented planes in different colors, and Figure 5b-1 shows the segmented planes of the selected area. Meanwhile, Figure 5c shows the 3D geometric modeling produced from the RANSAC algorithm described in the previous section, and Figure 5c-1 shows geometric modeling of the selected area. Geometric modeling can support modelers in enhancing the effectiveness and efficiency of as-built BIM, since it can precisely represent the boundaries and locations of building components. Consequently, the role of geometric modeling has a decisive effect on overall as-built modeling productivity with respect to both modeling accuracy and modeling time [14,15].

### 4.3. Implementation of As-Built BIM

As-built BIM performs three main tasks: geometric modeling, assignment of object categories and material properties, and setting of relationships between components. These tasks do not have to be sequential; the sequence will depend on the workflow [49]. The main focus of the present study was the geometric modeling of as-built BIM for Gangnam subway station. This section explains how as-built BIM is achieved based on 3D geometric modeling. It is shown that the boundary lines extracted from 3D geometric modeling can support the identification of the shape of 3D building components.

For Gangnam subway station, the traced boundaries from the geometric modeling and remaining points were converted to AutoCAD DXF (Drawing Exchange Format) and then imported to Autodesk Revit for as-build BIM. The unique aspects of this study in relation to other as-built BIM research [14,15] are the following: (1) the purpose of modeling—the as-built modeling was focused on applying the 3D underground cadastral system, not just for building modeling, but also to register underground properties according to underground cadastral maps; (2) the extent of modeling—the extent of the study area was large compared with previous studies; (3) the accuracy assessment of modeling—for use of as-built BIM to register an underground property into the cadastral system and, so, create a 3D underground parcel, accuracy assessment is a prerequisite.

**Figure 6:**
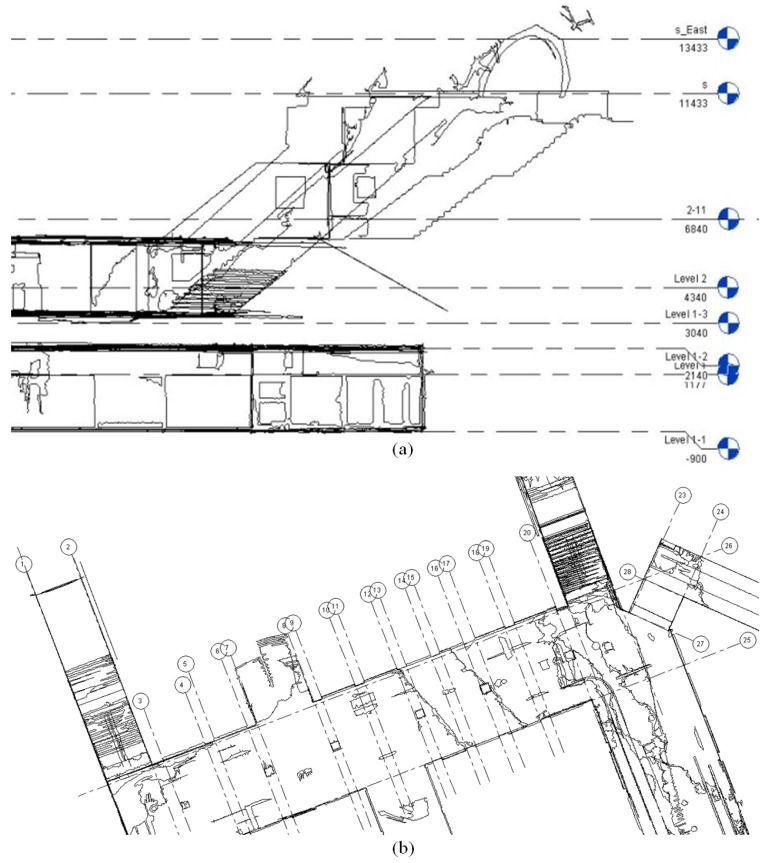
Level and grid line creation: (**a**) Created level line and (**b**) Created grid line.

**Figure 7:**
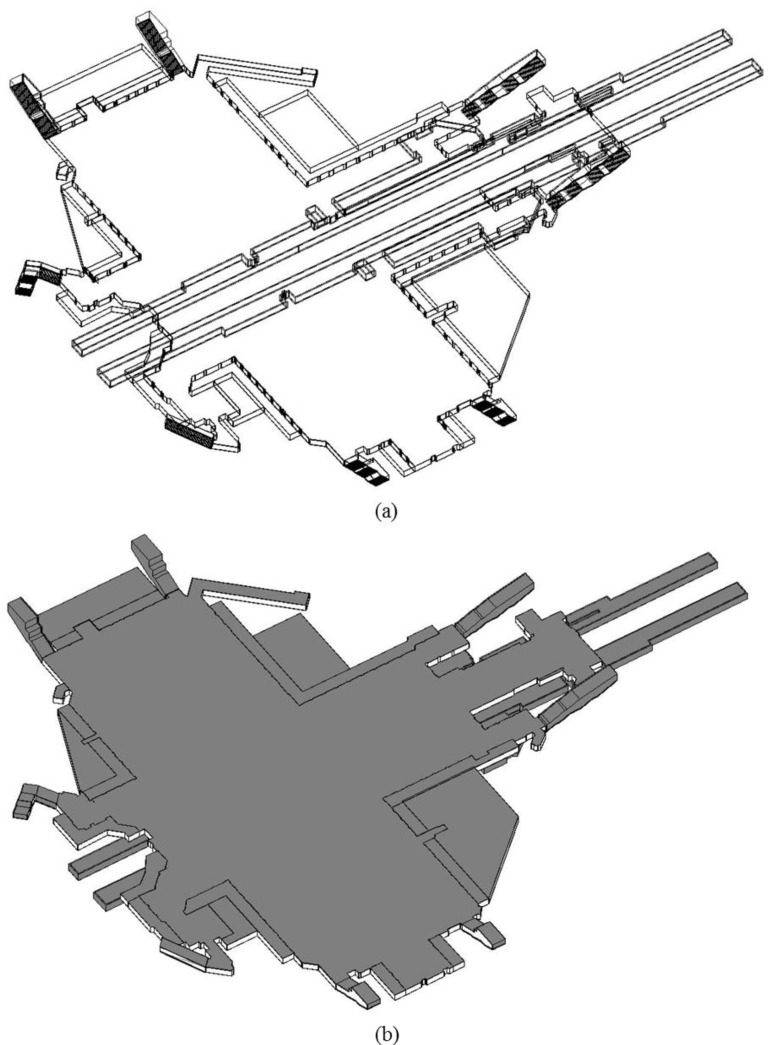
As-built BIM of Gangnam subway station: (**a**) Wire-frame type; (**b**) Solid type.

In this study, Autodesk Revit 2014, commercial BIM software, was used to conduct as-built modeling. In Revit, creation of BIM products is divided into three steps: (1) setting the level and grid line; (2) creating walls, floor, and ceiling; (3) detail modeling. The level and grid line creation is the very beginning step to create as-built BIM. In this study area, Gangnam subway station, a total of eight height levels were defined from the specific horizontal planes by considering floors, stairs, and ceiling. For the grid line creation, the numerous grid lines were created mainly by considering the wall components for each floor. Then, the floors and walls were created based on the setup level and grid line. Figure 6 illustrates the creation of the level and grid line. Finally, detail modeling, including a total of eight staircases and the limitation area, was conducted. Figure 7 represents the wire-frame and solid types of created as-built BIM of Gangnam subway station.

### 4.4. Accuracy Assessment of As-Built BIM Implementation

In order to apply as-built BIM of Gangnam subway station to the 3D underground cadastral system, horizontal and vertical accuracy assessment was conducted for the project site. Accuracy assessment includes three phases: (1) control-point surveying; (2) target-coordinate acquisition; and (3) accuracy assessment.

**Figure 8:**
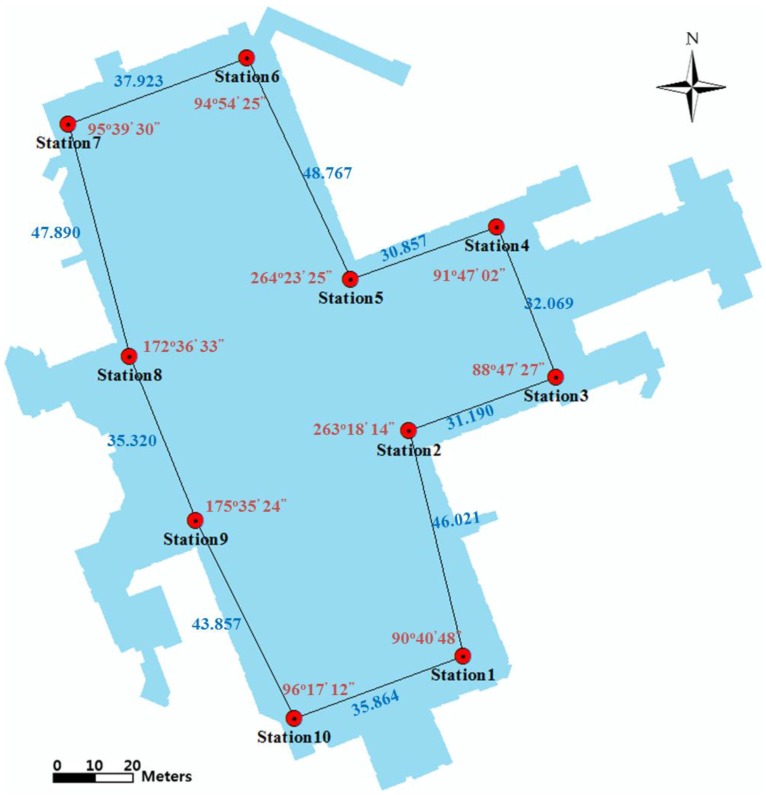
Distribution of control points and given traverse network.

In the control-point surveying phase, the closed traverse and leveling network with 10 control points is designed with consideration of the given underground environment. For the adjustment of the horizontal observations with the traverse network, observations were made of the total station, and the compass rule was applied. The angular misclosure of the traverse was −3″, and the linear misclosure was 0.007 m. For adjustment of the vertical observations, electronic digital/bar-code leveling was used; the misclosure of the observations was 0.00043 m. Table 2 summarizes the surveying equipment, and Figure 8 illustrates the distribution of the control points and given traverse network.

**Table 2:** Specifications of total station and electronic digital/barcode level.

Equipment	Specifications
Total Station	Model: GTS 9001 A, Topcon
	3D position accuracy: 3 mm at 50 m, 6 mm at 100 m
	Prism mode/linearity error: ±(2 mm + 2 ppm × D)
	Non-prism mode/linearity error: ±(5 mm)
Electronic Digital/Barcode Level	Model: Leica DNA 03
	Accuracy of electronic measurement: 0.3 mm (invar staffs)
	Resolution height measurement: 0.01 mm
	Compensator-setting accuracy: 0.3″
	Single measurement time: typically 3 seconds

**Figure 9:**
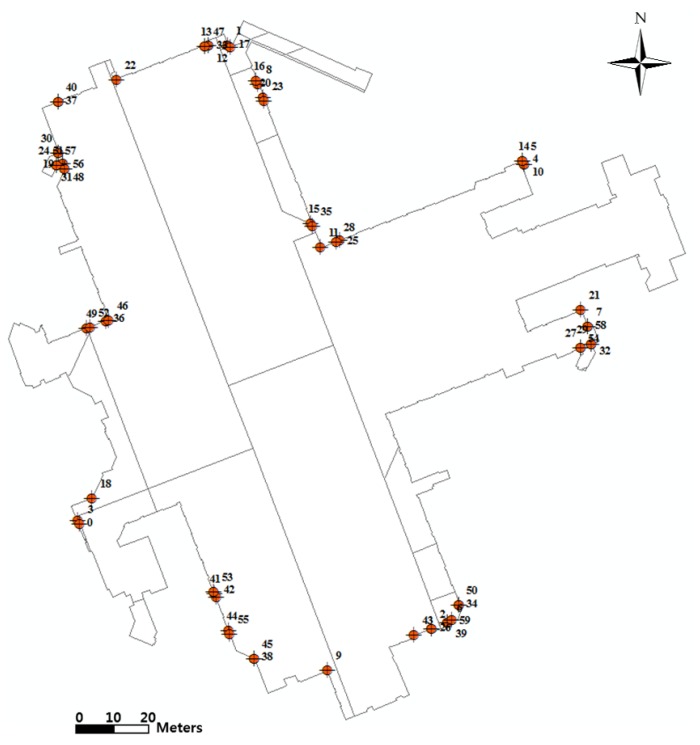
Distribution of 60 target points for accuracy assessment.

For accuracy assessment, the surveyed reference points and corresponding as-built modeling points have to be prepared. In the present case, a total of 60 target points were selected for the accuracy assessment for the 3D as-built BIM of Gangnam subway station. They are well-defined points such as corners of walls on the floor and ceiling, and the reference coordinates were acquired by way of radial survey of the total station based on the 10 3D control points described in the above. Figure 9 illustrates the distribution of the 60 target points.

In the accuracy assessment step, the quality of the 3D wire-frame model from the as-built BIM was evaluated based on the acquired target points. To that end, the Euclidean average distance error ($\delta_{avg}$) was assessed according to Equation (3):

(3) $$\delta_{avg}=\frac{1}{n}\sum_{i=1}^{n}\left|\text{R}a_{i}-\text{T}-b_{i}\right|$$

where $a_{i}$ is the *i*-th point vector in the 3D wire-frame model, $b_{i}$ is the corresponding point vector measured by total station, n is the number of point measurements, and R and T are the rotation and translation parameters, respectively, for 3D Helmert transformation (the scale was not considered in this comparison) [79]. The average error, thus calculated, indicated that the overall accuracy was 0.086 m. According to Korea cadastral surveying regulations, the acceptable error tolerance in terms of Euclidean average distance error is 10 cm for a digital cadastral map, which records the boundaries of a 2D surface parcel by a set of coordinates (x and y) [80]. Even though the calculated Euclidean average distance error should be used only for the 2D surface parcel, it showed that the proposed 3D underground cadastral maps offered a level of accuracy within the allowable error tolerance.

Additionally, the RMSE and the SAS were computed. The RMSE was calculated by Equation (4):

(4) $$RMSE=\sqrt{\frac{1}{n}\sum_{i=1}^{n}{\left(a_{i}^{t}-b_{i}\right)}^{2}}$$

where $a_{i}^{t}$ is the point transformed to total station coordinates. The calculated RMSE for Gangnam subway station was 0.095 m; each direction (*x*, *y*, and *z*) of the RMSEs are listed in Table 3. The SAS, defined as the spherical radius of a 90% probability sphere [81], was computed by Equation (5):

(5) $$SAS=0.833\;\times \;\left(RMSE_{x}+RMSE_{y}+RMSE_{z}\right)$$

as 0.129 m, which represents the positional accuracy of the two generated 3D wire-frame models at the 90% confidence level.

### 4.5. 3D Underground Cadastral Map

Visualization and documentation of a land parcel directly affect protection of land ownership and decision-making in land administration. However, in Korea, the current cadastral map represents only 2D parcel boundaries, without any additional information related to 3D property such as representation of the property boundary. For that reason, this paper proposes three types of cadastral maps: (1) isometric view of 3D underground cadastral map; (2) 2D surface parcel with footprints of 3D underground cadastral map; (3) 3D surface and 3D underground cadastral maps, based on as-built BIM.

The isometric view of the 3D underground cadastral map plays the key role in obtaining a better understanding of the 3D geometry of an underground parcel. The isometric view should support three types of underground parcel map: (1) 3D underground internal boundary from as-built BIM, which is related to the internal boundary of the underground property; (2) “3D underground construction parcel” boundary, which is related to the exterior boundary of the underground property; and (3) “3D underground legal space parcel” boundary, which is related to the safety or restricted zone for protection of the underground construction boundary according to the Urban Railroad Act’s ”Compensation Criteria for Underground Land Use for Urban Railroad”.

**Figure 10:**
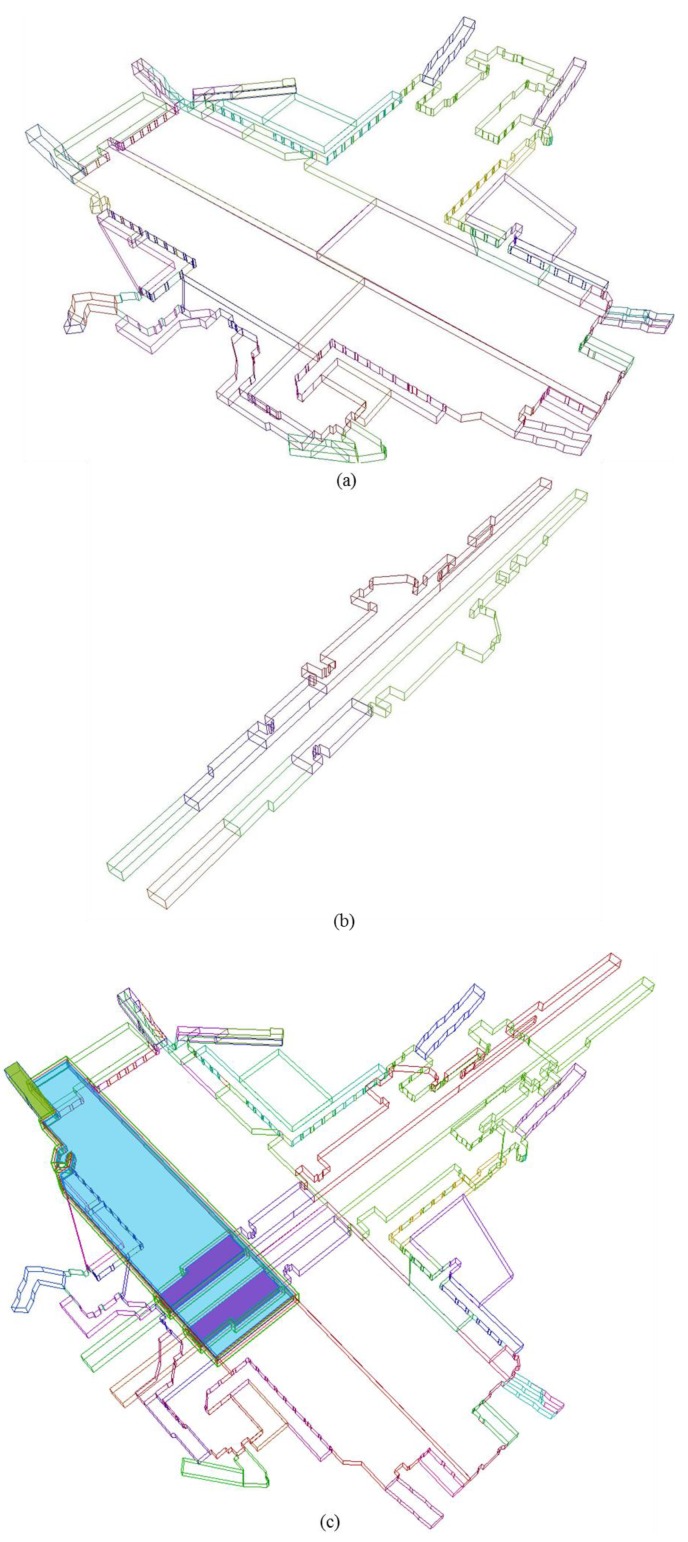

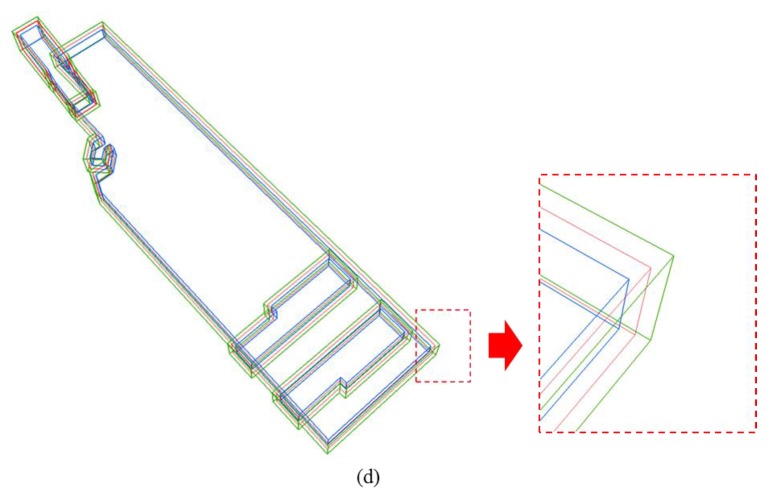
Iso metric view of 3D underground cadastral map in Gangnam subway station: (**a**) 3D underground construction parcel boundary of 1st underground floor: Gangnam underground shopping mall; (**b**) 3D underground construction parcel boundary of 2nd underground floor: subway station platform; (**c**) 3D underground construction parcel boundary of Gangnam subway station with highlight on particular underground parcels; and (**d**) 3D underground internal boundary (blue), 3D underground construction parcel boundary (red), and 3D underground legal space parcel boundary (green).

Figure 10 provides isometric views of the 3D underground cadastral map of Gangnam subway station: (a) 1st underground floor: Gangnam underground shopping mall; (b) 2nd underground floor: subway station platform; (c) Gangnam subway station with highlighted specific underground parcels; and (d) enlargement of specific underground parcels showing 3D underground parcel from as-built BIM (blue color), “3D underground construction parcel” boundary (red color) which attaches a 0.5 m thickness of wall, floor, and ceiling to the as-built BIM based on architectural drawing of Gangnam subway station, and “3D underground legal space parcel” boundary (green color) which attaches an additional 0.5 m thickness of ”protection layer” to the “3D underground construction parcel” boundary by ”Administrative Rules of the Railroad Construction Act on Compensation Criteria for the Underground Land Use for Railroad Construction“ in Korea.

The area and volume of the underground shopping mall and subway station platform were calculated as attributes of the 3D underground properties. In the case of the underground shopping mall, the area and volume were 14,838.8 m^2^ and 38,580.8 m^3^, and in the case of the subway station platform, 3679.7 m^2^ and 15,086.9 m^3^ respectively.

**Figure 11:**
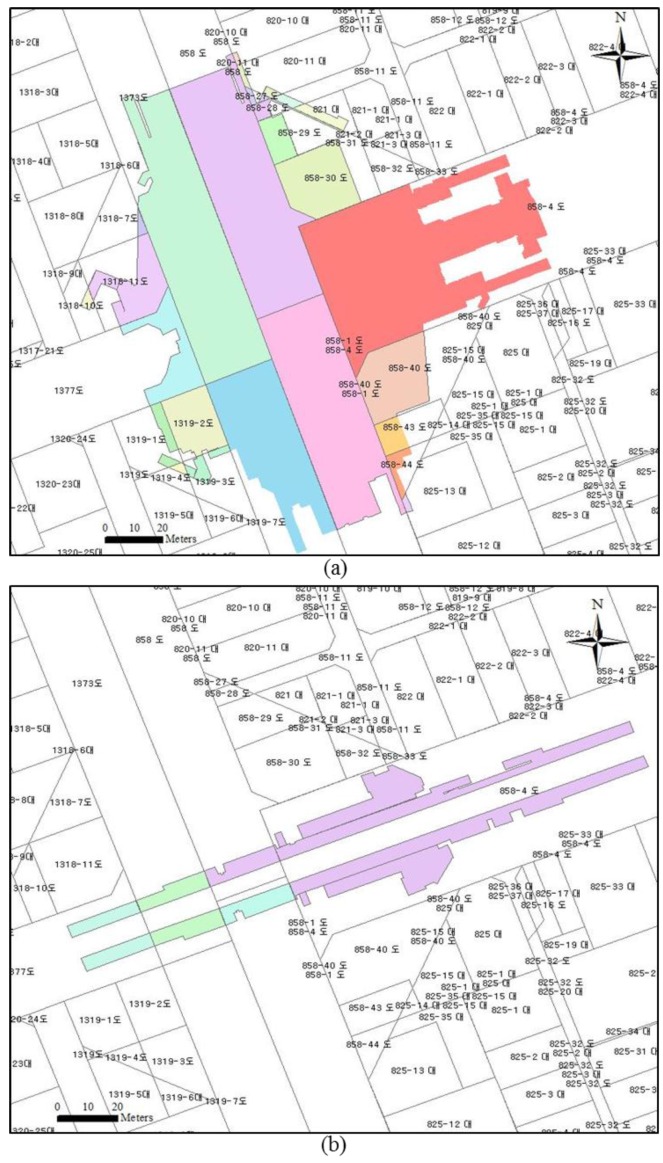
2D surface parcel with footprints of 3D underground cadastral map in Gangnam subway station: (**a**) 1st underground floor: Gangnam underground shopping mall; and (**b**) 2nd underground floor: subway station platform.

In the case of the 2D surface parcel with footprints of the 3D underground cadastral map, the 3D underground subway station was divided into several parts according to the surface parcel boundaries in order to confirm the relationship between the 2D surface parcel and the 3D underground parcel. The Gangnam underground shopping mall is occupied by 26 surface parcels, of which 22 are owned by Seoul Metropolitan Government. The land categories are designated as ”road”. The remaining four parcels are privately owned, the land category of which is designated ”building site”. The ownership of these four private parcels had to be registered by sectional superficies to protect it. As another example, the subway station platform has been occupied by five surface parcels owned by Seoul Metropolitan Government, and the land categories are designated ”road”. Figure 11 illustrates the 2D footprint with 3D underground cadastral map. Table 4 provides detailed information on the relationship between the 2D surface parcel and the 3D underground parcel.

**Table 3:** Accuracy assessment results (unit: meters).

Point ID	Error Vector			Error	Point ID	Error Vector			Error
	X	Y	Z			X	Y	Z	
1	0.119	−0.054	0.091	0.160	31	−0.049	−0.012	−0.088	0.101
2	0.021	0.007	0.141	0.143	32	−0.076	−0.021	−0.026	0.083
3	−0.067	−0.131	−0.085	0.170	33	−0.022	0.027	0.051	0.062
4	0.002	0.013	−0.181	0.181	34	−0.012	0.010	0.045	0.048
5	0.005	0.018	−0.174	0.175	35	0.012	−0.017	0.050	0.054
6	−0.036	0.046	0.112	0.126	36	−0.048	0.011	−0.071	0.086
7	0.078	0.106	−0.087	0.158	37	0.063	−0.001	0.016	0.065
8	−0.049	0.031	0.096	0.112	38	−0.010	−0.044	0.006	0.046
9	−0.031	0.102	0.053	0.119	39	−0.003	0.032	0.042	0.052
10	0.010	0.033	−0.138	0.142	40	0.066	0.002	−0.045	0.080
11	0.095	−0.042	0.047	0.115	41	−0.044	0.017	−0.004	0.047
12	−0.014	0.008	0.103	0.104	42	−0.030	−0.023	0.003	0.038
13	−0.010	−0.004	0.102	0.102	43	0.033	0.009	0.033	0.047
14	0.008	0.028	−0.134	0.137	44	−0.033	0.031	−0.002	0.046
15	0.021	−0.007	0.097	0.099	45	−0.008	−0.037	−0.053	0.066
16	0.012	−0.003	0.096	0.097	46	−0.010	0.025	−0.070	0.075
17	−0.024	0.004	0.095	0.098	47	−0.013	0.008	0.032	0.036
18	0.009	−0.010	−0.142	0.142	48	0.013	−0.026	−0.073	0.078
19	−0.091	0.044	−0.030	0.106	49	−0.005	0.015	−0.070	0.072
20	−0.001	−0.003	0.085	0.086	50	−0.017	−0.002	−0.058	0.060
21	0.008	−0.090	−0.020	0.093	51	0.001	−0.035	−0.061	0.070
22	0.008	−0.038	0.078	0.087	52	0.015	0.011	−0.066	0.068
23	−0.016	0.000	0.084	0.086	53	0.029	−0.021	−0.007	0.037
24	0.033	0.058	−0.095	0.116	54	−0.020	−0.011	0.007	0.024
25	0.012	−0.051	0.062	0.081	55	−0.006	0.017	−0.006	0.019
26	0.068	−0.013	0.047	0.084	56	0.008	−0.019	0.000	0.021
27	−0.001	0.014	−0.115	0.116	57	0.005	−0.030	−0.022	0.038
28	0.001	−0.044	0.059	0.074	58	0.003	0.022	−0.010	0.025
29	−0.082	−0.023	−0.015	0.086	59	0.043	0.006	0.035	0.056
30	0.028	0.056	0.030	0.069	60	−0.002	0.006	0.121	0.121
Average Error						-	-	-	0.086
RMSE						0.039	0.038	0.078	0.095
SAS						-	-	-	0.129

**Table 4:** Relationship between 2D surface parcel and 3D underground parcel.

2D Surface Parcel		3D Underground Parcel					
Parcel Number	Land Category	Underground Parcel Number	Utiliza-tion	Ownership	Right	Area (m^2^)	Volume (m^3^)
858-4	Road	858-4-1	US	SMG	FO	3475.3	9035.8
858-40	Road	858-40-1	US	SMG	FO	610.2	1586.4
858-43	Road	858-43-1	US	SMG	FO	116.4	302.7
858-44	Road	858-44-1	US	SMG	FO	82.7	215.1
825-13	Building site	825-13-1	US (Exit)	Private land	SS	21.9	56.9
858-1	Road	858-1-1	US	SMG	FO	1956.2	5086.1
1374	Road	1374-0-1	US	SMG	FO	1380.5	3589.2
1319-3	Road	1319-3-1	US	SMG	FO	107.1	278.5
1319-4	Road	1319-4-1	US	SMG	FO	23.2	60.3
1319-1	Road	1319-1-1	US	SMG	FO	119.3	310.3
1319-2	Road	1319-2-1	US	SMG	FO	377.4	981.1
1377	Road	1377-0-1	US	SMG	FO	430.7	1119.8
1318-10	Road	1318-10-1	US	SMG	FO	33.9	88.0
1318-11	Road	1318-11-1	US	SMG	FO	442.9	1151.5
1318-7	Road	1318-7-1	US	SMG	FO	29.1	75.6
1373	Road	1373-0-1	US	SMG	FO	2377.6	6181.8
858	Road	858-0-1	US	SMG	FO	2220.2	5772.5
820-10	Building site	820-10-1	US (Exit)	Private land	SS	15.0	39.0
820-11	Building site	820-11-1	US (Exit)	Private land	SS	51.7	134.4
858-11	Road	858-11-1	US	SMG	FO	34.8	90.4
821	Building site	821-0-1	US (Exit)	Private land	SS	80.4	208.9
858-29	Road	858-29-1	US	SMG	FO	200.5	521.4
858-30	Road	858-30-1	US	SMG	FO	565.5	1470.3
858-28	Road	858-28-1	US	SMG	FO	51.8	134.6
858-32	Road	858-32-1	US	SMG	FO	10.6	27.5
858-27	Road	858-27-1	US	SMG	FO	24.1	62.6
1373	Road	1373-0-2	SP	SMG	FO	382.4	1567.9
1377	Road	1377-0-2	SP	SMG	FO	348.8	1429.9
858	Road	858-0-2	SP	SMG	FO	198.0	811.6
858-1	Road	858-1-2	SP	SMG	FO	205.7	843.3
858-4	Road	858-4-2	SP	SMG	FO	2544.9	10434.2

US: Underground Shopping Mall; SP: Subway Station Platform; SMG: Seoul Metropolitan Government; FO: Full Rights of Ownership; SS: Sectional Superficies.

**Figure 12:**
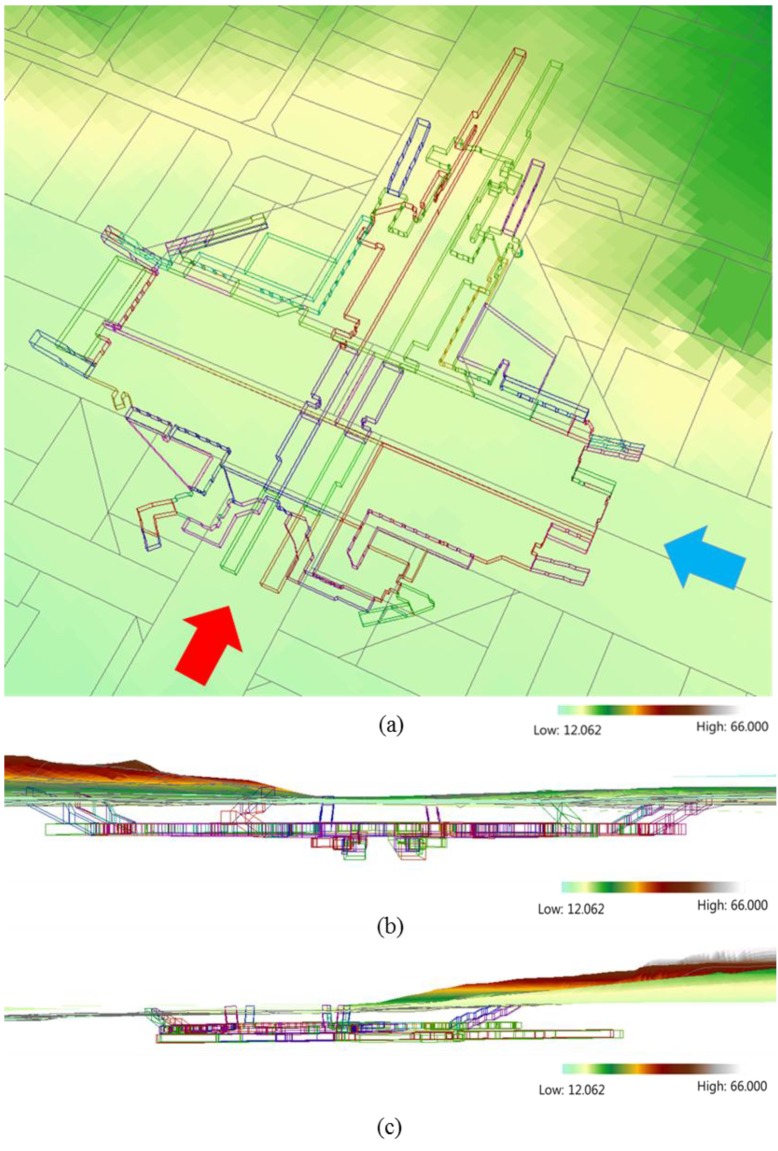
3D surface and 3D underground cadastral map (unit: meters): (**a**) top view of 3D underground parcel with transparency effect; (**b**) Front view of 3D underground property (direction of red arrow on Figure 12a); and (**c**) Side view of 3D underground property (direction of blue arrow on Figure 12a).

In this paper, a 3D surface and 3D underground cadastral map is proposed. In order to create the 3D surface, a 1:1000 digital map containing ±0.3 m of vertical error is used [82]. Then, this map is integrated with 2D surface parcel boundaries to produce height information on the 3D underground cadastral property. It is possible to represent the relative height, which is the vertical distance between the 3D surface and 3D underground cadastral property. The relative height is determined by calculating the distance between all corner points on the ceiling of 3D underground construction parcel and 3D underground legal parcel, and their corresponding points of 3D surface. This relative height information is critical to the verification of the range of underground ownership with respect to the surface height. It can establish rights, responsibilities, and restrictions related to the underground property by preventing the conflict of ownership right, protecting the developed underground facility, and facilitating public use. Figure 12 illustrates a 3D surface and 3D underground cadastral map: (a) is the top view of the 3D underground cadastral map by the transparency effect; (b) is the front view of the 3D underground property (see the red arrow in Figure 12a); and (c) is the side view of the 3D underground property (see the blue arrow in Figure 12a).

The three types of 3D underground cadastral maps can clearly represent the status of 3D underground property, and so, by provision of sufficient geometric information, can support registration of legal information including rights, responsibilities, and restrictions.

## 5. Conclusions

The proposed method represents a new concept in the registration of underground property in relation to a 3D underground cadastral system. Specifically, the method represents accurate and diverse spatial information on 3D underground property for legitimization of registration of rights, responsibilities, and restrictions, as well as for prevention or adjudication of vertical land disputes, proper land compensation, and improvement of the taxation system. The major contribution of this study is its implementation, for the first time, of a 3D underground cadastral system in a real-world setting: the Gangnam subway station in Seoul, Korea.

In order to fulfill its intended function, the 3D underground cadastral system requires effective and efficient indoor mapping technology; therefore, the authors chose a terrestrial laser scanner for relevant indoor mapping, which utility has been extensively employed in the as-built BIM arena. The proposed method consists of four processing steps: (1) geometric modeling of underground construction components; (2) as-built BIM in Revit software; (3) accuracy assessment of as-built BIM; (4) production of 3D underground cadastral maps based on as-built BIM: an isometric view of the 3D underground cadastral map, a 2D surface parcel with footprints of the 3D underground cadastral map and the 3D surface and 3D underground cadastral maps. In the present study, the proposed method was, as noted above, applied to a real-world underground infrastructure, Gangnam subway station in Seoul, Korea. The 3D underground cadastral maps based on indoor mapping for as-built BIM can, by clearly identifying the underground physical situation, facilitate better decision-making in the management, maintenance, and development of underground property. While working on the implementation, we could confirm the effectiveness and efficiency of the proposed indoor mapping method for as-built BIM and, eventually, 3D underground cadastral mapping. Simple polylines, which are the boundaries of the underground property, were extracted from the huge-size raw point cloud data, which BIM software might not be able to handle or even to import. We could also prove that geometric modeling can be a powerful solution to the problem of producing accurate 3D underground cadastral mapping in an efficient manner.

In order to apply the method to the 3D underground cadastral system, accuracy assessment was conducted based on target points acquired by total station. The result showed that the Euclidean average distance error was 0.086 m and the RMSE 0.095 m. This error tolerance, based on the Euclidean average distance error and according to the Korea cadastral surveying accuracy standard, was acceptable within 10 cm for a 2D digital cadastral map. Overall, the proposed indoor mapping for as-built BIM was proven to be an effective solution satisfying the requirements of the 3D underground cadastral system.

The authors recognize that the proposed new framework for 3D underground cadastral system will require additional modification for real-world applications. Specifically, several issues have to be resolved, not only on the technical side but also on the administration and law side, such as the definition of ”3D underground cadastre”, including the numbering system of underground parcel ID’s, underground land categories, area and volume, and the reestablishment of ”the scope of justifiable profit“ in the Civil Law to guarantee underground ownership. Finally, in order to improve the usability and potential value of the 3D underground cadastral system, semantic information on 3D underground properties has to be provided in proven legitimate and efficient ways. In this regard, International Property Measurement Standards [83], which represent an important international initiative to ensure that measurement of various types of properties is performed in a consistent way, need to be adopted to ensure efficient and effective land management service.
